# A Novel ELISA Assay for the Detection of Anti-Prothrombin Antibodies in Antiphospholipid Syndrome Patients at High Risk of Thrombosis

**DOI:** 10.3389/fimmu.2021.741589

**Published:** 2021-09-08

**Authors:** Mathivanan Chinnaraj, Vittorio Pengo, Nicola Pozzi

**Affiliations:** ^1^Edward A. Doisy Department of Biochemistry and Molecular Biology, Saint Louis University School of Medicine, St. Louis, MO, United States; ^2^Thrombosis Research Laboratory, Department of Cardiac-Thoracic-Vascular Sciences and Public Health, University of Padova, Padua, Italy; ^3^Arianna Foundation on Anticoagulation, Bologna, Italy

**Keywords:** antiphospholipid antibody syndrome, autoimmunity, acquired coagulation disorders, lipid-protein interaction, single-molecule biophysics, COVID-19

## Abstract

Autoantibodies targeting prothrombin (aPT) can be found in antiphospholipid syndrome (APS) patients. However, their detection has proven difficult to standardize. Here, we developed a new ELISA assay to improve the identification of aPT and compared its performance with currently available anti-phosphatidylserine/prothrombin antibodies (aPS/PT) and autoantibodies targeting prothrombin bound to the plastic plate (aPT-A) assays using a cohort of 27 APS patients at high risk of thrombosis. We generated a novel prothrombin variant, ProTS525A-Biot, carrying an artificial tag at the C-terminus suitable for site-specific biotinylation and added the mutation S525A to improve stability. ProTS525A-Biot was immobilized to neutravidin-coated plates at the desired density and with a defined orientation, i.e., pointing the N-terminal fragment-1 toward the solvent. Antibodies against ProTS525A-Biot (aPT-Bio) were found in 24 out of 27 triple-positive APS patients (88%). When compared to aPS/PT and aPT-A, aPT-Bio showed an excellent linear correlation with aPS/PT (R^2 =^ 0.85) but not with aPT-A (R^2 =^ 0.40). Since aPS/PT but not aPT-A are an emerging biomarker of thrombosis in APS, this method may find utility for detecting pathogenic aPT in APS but also other prothrombotic conditions such as COVID-19.

## Introduction

Antiphospholipid syndrome is a debilitating condition characterized by vascular thrombosis in the presence of antiphospholipid antibodies (aPL), which persist >12 weeks in patients’ plasma (1). Among aPL, anti-prothrombin antibodies (aPT) are believed to be clinically relevant since they are linked to thrombosis (2). Currently, aPT are detected using two methods (3). First, prothrombin is directly immobilized onto a hydrophilic plastic plate, in which case aPT are named aPT-A. Alternatively, prothrombin is bound, in the presence of calcium ions, to plastic wells pre-coated with phosphatidylserine (PS), in which case aPT are referred as to anti-phosphatidylserine/prothrombin antibodies (aPS/PT). Beyond the technical aspects, this distinction has strong clinical value since aPS/PT but not aPT-A are the ones often found in APS patients at high risk of thrombosis (3, 4), arguing for a potential pathogenic role in the onset and progression of APS disease.

Despite recent advances in analytical methods, detection of aPT, and especially aPS/PT, has proven difficult to standardize because of the transient nature of the phospholipid-bound complex, which requires calcium ions, and the variable source/purity of phospholipids and antigen. Furthermore, even though it is assumed that aPS/PT interact with prothrombin, they may react against PS and other plasma proteins capable of interacting with PS (5). The aim of this study was to develop an ELISA assay to improve the identification of aPT in correlation with thrombosis.

## Materials and Methods

### Protein Expression and Purification

All prothrombin variants used in this study were produced in Expi293 cells (Thermo Fisher Scientific, USA) in the presence of vitamin K and purified as described before (6). Briefly, the cDNA of human prothrombin (ProTWT, UniProtKB P00734) modified to include an epitope for the HPC4 antibody (7) at the C-terminus was cloned into a pDEST40 expression vector (Life Technologies, Inc.) and sequenced verified by Genewiz. Differently from ProTWT, ProTS525A-Biot carries 1) an extended C-terminal peptide sequence ^1^GGGSGLNDIFEAQKIEWHE^19^ inserted after the HPC4-purification tag that can be specifically biotinylated *in vitro* by the biotin ligase enzyme (Avidity, USA) at the lysine residue 14 and 2) a single point mutation, namely the catalytic serine (S) 525 was substituted to alanine (A). Genetic engineering of the original cDNA was attained by PCR using the Quickchange Lighting kit (Agilent) and appropriate primers (Integrated DNA Technologies). Transfection of Expi293 cells was performed using Lipofectamine 3000 (ThermoFisher, USA) following manufacturers’ instructions. Selection of stably expressing clones was initiated 24 hours after transfection using the antibiotic geneticin (G-418, GoldBio, USA). Prothrombin secreted in the media was purified in three sequential steps: immunoaffinity, ion-exchange chromatography and size exclusion chromatography, as detailed elsewhere (6). A key step for obtaining highly pure recombinant protein was to load the immunopurified material into a heparin column (HiTrap Heparin HP 1 ml, Cytiva) before passing the solution into a Q-column (HiTrap Q HP 1 ml, Cytiva). This is because prothrombin fragments generated during protein production, storage, and purification (namely thrombin and prethrombin-2), but not intact prothrombin, bind to heparin. *In vitro* biotinylation was performed using BirA500 biotin-protein ligase reaction kit (Avidity). Incorporation of biotin was verified using QuantTag Biotin Quantitation Kit (Vector Laboratories).

### Biochemical and Biophysical Studies

Surface plasmon resonance (SPR), kinetic studies and single-molecule Förster resonance energy transfer (smFRET) experiments with ProT120/478 and ProT120/478/S525A-Biot labeled with AlexaFluor-555 and AlexaFluor-647 maleimide were performed as described before (6, 8–10). Briefly, confocal smFRET data were collected on freely diffusing molecules using a MicroTime 200 microscope (PicoQuant) and analyzed using the MatLab based software PAM (11) to generate FRET histograms from hundreds of events. smFRET-total internal reflection fluorescence (TIRF) experiments were carried out with an objective-type TIRF microscope, essentially as described elsewhere (12, 13). Briefly, glass coverslips coated with polyethylene glycol (PEG)-biotin (MicroSurfaces, USA) with additional coverage of 1% Tween-20 to prevent protein sticking to glass coverslips were treated with neutravidin followed by addition of ProT120/478/S525A-Biot. The sparsely covered surface resulting from immobilization of ProT120/478/S525A-Biot was then imaged at 32 frames per second using Andor iXon EMCCD camera in Tris 20 mM, pH 7.4, 145 mM NaCl, 5 mM CaCl_2_, 0.01% Tween-20 supplemented with Trolox (2 mM) and glucose scavenging system (0.8% w/v D-glucose, 1 mg/ml glucose oxidase, and 0.04 mg/ml catalase). Data were processed using IDL script available from the Ha lab at http://ha.med.jhmi.edu/resources/. Traces were extracted using MatLab, then fitted to a two state Hidden Markov Model (HMM) model using vbFRET (14).

### Plasma Samples and IgGs

Collection of citrated plasma and purification of IgGs was described earlier (8). Briefly, venous blood was collected at the University of Padua in 3.8% sodium citrate (9:1) and centrifuged twice at 2000xg for 15 min at 4°C. Plasma was stored in 25 µL aliquots at -80°C ready for individual use. All the patients in this study participated in the TRAPS trial and gave their written informed consent to utilize their stored residual plasma in this study. APS was diagnosed according to the Sydney Criteria (15). Total IgG extracts were purified using Protein G spin columns (ThermoFisher, USA) followed by size exclusion chromatography to obtain pure IgG monomers. Each preparation was >98% pure as judged by sodium dodecyl sulfate–polyacrylamide gel electrophoresis. The IgG concentration was determined by reading at 280 nm with a molar extinction coefficient of 1.00 M^-1^ cm^2^.

### ELISA Assays

Neutravidin (3100, Thermo Fisher Scientific, USA) was resuspended in water and its concentration determined by reading absorbance at 280 nm using a molar extinction coefficient of 1.66 M^-1^ cm^2^. For the detection of aPT-Biot, one hundred microliters of a solution of neutravidin solubilized in 0.1M sodium bicarbonate pH 9.6 were added to a Nunc MAXISORP plate (MilliporeSigma, USA) and incubated overnight at 4°C. After washing three times with 200 µl/well of 20 mM Tris pH 7.4, 145 mM NaCl, 5 mM CaCl_2_, Tween 20 0.02% (TBS-T), wells were blocked with 200 µl of 20 mM Tris pH 7.4, 145 mM NaCl, 5 mM CaCl2, 1% BSA (TBS-B) for 60 min at room temperature. One hundred microliters of ProTS525A-Biot or ProTS525A at the desired concentration were incubated for 60 min at room temperature before repeating the washing step. Next, one hundred microliters of plasma (1:100 v/v dilution) or IgGs prepared in TBS-B were added to each well and incubated for 60 minutes at room temperature (18-22°C). Plates were washed 3 times TBS-T before adding 100 μl of 1:10,000 dilution of peroxidase conjugated anti-human IgG, γ-chain specific (A6029, MilliporeSigma, USA) for 60 minutes at room temperature. Finally, plates were washed three more times with TBS-T and then incubated with 100 μl of 1-Step 3,3’,5,5’-Tetramethylbenzidine (TMB) Liquid Substrate (34028, ThermoFisher, USA). After 30 minutes, the colorimetric reaction was quenched with 100 μl of TMB-stop solution. The optical density at 450 nm was recorded using a SPARK microplate reader (TECAN, Austria). Statistical analysis between the groups was performed using t-tests in Prism 9.0 (*** < 0.001). aPT-A and aPS/PT were detected using procedures published previously (8).

## Results

Recent studies from our laboratory have documented that aPS/PT, despite being heterogeneous, preferentially bind to the N-terminal portion of prothrombin, also known as fragment-1 (8). Because of this finding, we hypothesized that detection of aPT in correlation with thrombosis might be possible by using a prothrombin derivative that can be immobilized to ELISA plates at the desired density and with a defined orientation, i.e., pointing the N-terminal fragment-1 toward the solvent. To this goal, we created ProTS525A-Biot. ProTS525A-Biot is a catalytically inactive version of prothrombin, which is specifically biotinylated at the C-terminus and can therefore be immobilized onto neutravidin coated plates at the desired density and orientation. Since prothrombin spontaneously converts to thrombin over time, thus resulting in fragment-1 and prethrombin-1 (16), the S->A substitution was chosen to improve the long-term storage and stability of the protein at physiological pH and ionic strength concentrations.

**Figure 1:**
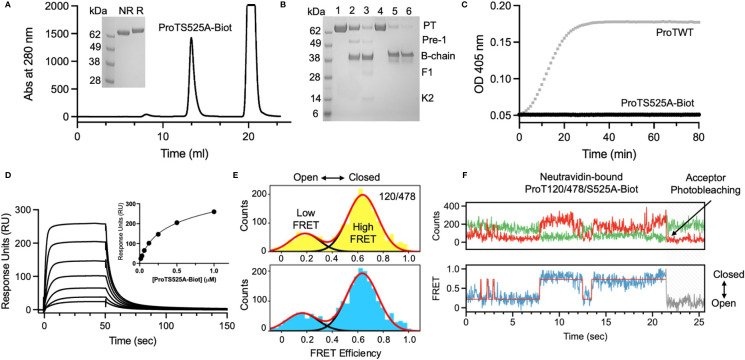
Structural and functional characterization of ProTS525A-Biot. **(A)** Size exclusion chromatography (SEC) of ProTS525A-Biot on a Superdex S200 after biotinylation reaction. ProTS525A-Biot elutes at 13 ml. Excess of ATP needed for the biotinylation reaction is eluted with the void volume (20 ml). **(B)** Conversion of ProTWT (0.1 mg/ml, 1.4 µM, lanes 1,2 and 3) and ProTS525A-Biot (0.1 mg/ml, 1.4 µM, lanes 4,5 and 6) to thrombin by prothrombinase complex (fVa/fXa/POPC : POPS, 10 nM/0.4 nM/25 µM) in 150 mM NaCl, 20 mM Tris, 5 mM CaCl_2_ monitored by gel electrophoresis. Following addition of prothrombinase complex (t=0, lanes 1 and 4), samples (40 µl) were quenched at 5 (lanes 2 and 5) and 10 minutes (lanes 3 and 6) with 10 µl of NuPAGE LDS buffer containing β-mercaptoethanol as the reducing agent and 20 mM EDTA. The samples were processed by NuPAGE Novex 4 –12% Bis-Tris protein gels run with MES buffer. Gels were stained with Coomassie Brilliant Blue R-250. Proteolytic fragments are indicated: prothrombin (PT), prethrombin-1 (Pre-1), B-chain, Fragment-1 (F1) and Kringle-2 (K2). Note how the autoproteolytic fragments Pre-1 and F1 are visible only in ProTWT but not in ProTS525A-Biot, which is catalytically inactive. Also note how the B-chain of ProTS525A-Biot has slower electrophoretic mobility compared to the B-chain of ProTWT due to the presence of the C-terminal biotinylated tag. **(C)** Conversion of ProTWT (25 nM, gray line) and ProTS525A-Biot (25 nM, black line) to thrombin by prothrombinase complex (fVa/fXa/POPC : POPS, 2.5nM/2.5pM/20µM) monitored using a continuous chromogenic assay (6). **(D)** Binding of ProTS525A-Biot (0.015-1 µM) to negatively charged liposomes (100 nm diameter extruded LUV made of POPC : POPS 80:20 molar ratio) monitored by SPR. **(E)** Confocal smFRET experiments of ProT120/478 (top) and ProT120/478/S525A-Biot (bottom) documenting similar distribution between closed (high FRET) and open (low FRET) forms in solution, with a ~80:20 ratio. **(F)** smFRET-TIRF obtained by immobilizing ProT120/478/S525A-Biot onto a neutravidin coated coverslips showing real-time dynamic exchange between closed (high FRET) and open (low FRET) conformations. Representative anticorrelated changes in AlexaFluor 555 (top panel, green) and AlexaFluor 647 (top panel, red) intensities and corresponding FRET changes (bottom panel). The red line in the bottom panel represents HMM fit to a 2-state model obtained using vbFRET (14). Also shown is a single photobleaching event of the acceptor dye occurring very rapidly at 21.4 sec, indicative of a single prothrombin molecule.

ProTS525A-Biot was expressed in Expi293 cells in the presence of vitamin K. Size exclusion chromatography (SEC) after *in vitro* biotinylation reaction revealed that that the protein is highly pure and monomeric (**Figure 1A**). To confirm functional integrity and the presence of the S525A mutation, the conversion ProTS525A-Biot to thrombin by the prothrombinase complex was monitored by gel electrophoresis (**Figure 1B**) and by a chromogenic assay (6) (**Figure 1C**), respectively. As expected for a structurally intact yet inactive enzyme, ProTS525A-Biot was properly converted to thrombin by the prothrombinase complex (**Figure 1B**), but the resulting enzyme displayed no catalytic activity (**Figure 1C**). Given the importance of the N-terminal region of prothrombin in the context of aPS/PT binding, we performed additional SPR binding experiments using immobilized negatively charged liposomes to assess whether glutamic acids underwent proper γ-carboxylation. As shown in **Figure 1D**, binding of ProTS525A-Biot to liposomes was concentration dependent and saturable. The affinity constant calculated by plotting the response unit at equilibrium *versus* [ProTS525A-Biot] yielded a K_d_=0.23 ± 0.10 µM (inset, **Figure 1D**). This value is close to previously reported values obtained for ProTWT under the same experimental conditions (8), confirming structural and functional integrity of the Gla-domain.

We have previously shown that prothrombin is a very dynamic molecule, adopting closed and open forms in solution (6, 10). To elucidate whether ProTS525A-Biot retains the same dynamic properties of ProTWT when free and bound to neutravidin, we performed confocal (**Figure 1E**) and TIRF-based smFRET experiments (**Figure 1F**), respectively. Based on previous studies, we labeled positions 120 in kringle-1 and 478 in the serine protease domain with the FRET pair AlexaFluor 555/647. We found that ProT120/478/S525A-Biot, like ProT120/478, interconverted between closed (high FRET) and open (low FRET) forms in solution (**Figure 1E**). Remarkably, a similar behavior was observed when ProT120/478/S525A-Biot was bound to neutravidin, documenting that the engineered tag, active site mutation and immobilization strategy do not affect the structural and dynamic properties of the antigen (**Figure 1F**).

**Figure 2:**
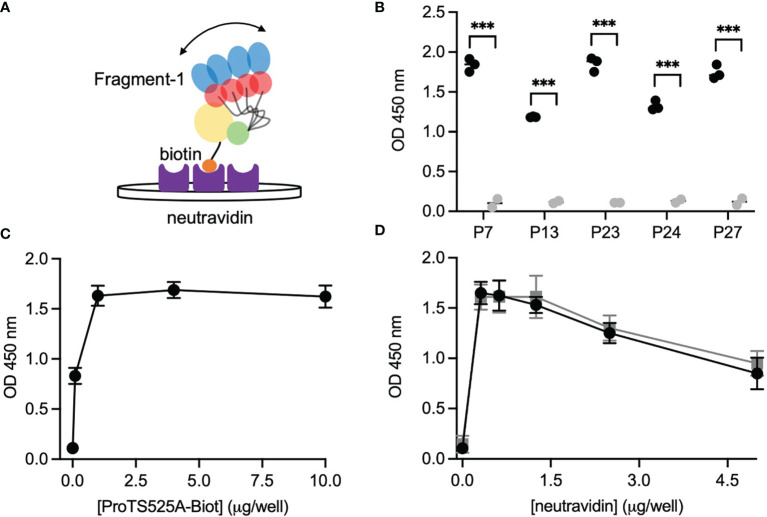
Design and validation of the new ELISA assay. **(A)** Cartoon showing the immobilization scheme of ProTS525A-Biot onto neutravidin coated plates and mobility of fragment-1 swinging between closed and open states documented by smFRET. **(B)** Control experiments using biotinylated (black) and non-biotinylated (gray) ProTS525A. Statistical analysis between the groups was performed using t-tests in Prism 9.0 (*** < 0.001). Optimization experiments with different concentrations of ProTS525A-Biot **(C)** and neutravidin **(D)**. Total IgGs were purified as described before (8) and used at a concentration of 75 µg/ml. Representative results obtained with P27 are shown in **(C)**, whereas results with P7 (black) and P27 (gray) were chosen for **(D)**.

After having established that ProTS525A-Biot retained identical structural and functional properties compared to ProTWT, we tested whether aPT could recognize ProTS525A-Biot bound to neutravidin plates (**Figure 2**). We initially used total IgG extracts that were purified from 5 APS patients with high titers of aPT-A and aPS/PT. The laboratory profile of these patients is shown in **Table 1**. Neutravidin at 1 µg/well was immobilized onto hydrophilic plates and, after extensive washing, 100 µl of ProTS525A-Biot at a concentration of 10 µg/ml was added. Non-biotinylated ProTS525A was used as a negative control. The results in **Figure 2B** show that aPT strongly reacted against ProTS525A-Biot. To differentiate these autoantibodies from aPT-A and aPS/PT, we called them aPT-Biot. Importantly, we observed no reactivity against neutravidin that was incubated with non-biotinylated ProTS525A. This result validates our design and documents no cross-reactivity of aPT against neutravidin. Intra and inter-assay variability were assessed by independently repeating the ELISA experiment three times, using IgG samples from 17 patients (**Table 2**). Intra-assay variability, calculated on triplicates of the same sample, was < 5%; inter-assay variability was, on average, 10% (min 3%, max 17%).

**Table 1:** Laboratory profile of the 27 APS patients (P01-27) and 5 healthy controls (C01-C05) used in the study.

	aCL	aβ_2_GPI	aPS/PT	aPT-A	aPT-Biot
P01	0.07	*0.14	1.06	0.25	0.76
P02	0.28	0.80	0.26	0.07	0.03
P03	1.01	2.32	3.48	1.78	2.60
P04	0.59	0.87	2.85	0.61	1.71
P05	0.41	0.65	0.22	0.05	0.34
P06	1.55	1.85	2.05	0.08	0.89
P07	0.28	0.13	3.23	2.19	2.67
P08	1.90	1.68	0.94	0.00	1.54
P09	0.93	0.40	3.34	2.08	2.34
P10	0.36	1.09	1.12	0.00	0.77
P11	0.36	0.71	0.50	0.00	0.32
P12	0.97	1.70	0.40	0.00	0.66
P13	1.55	2.43	2.04	1.00	1.28
P14	1.11	1.74	2.73	0.50	1.59
P15	1.31	1.35	0.36	0.01	0.19
P16	0.41	0.37	1.34	0.06	0.51
P17	1.26	2.14	2.04	0.06	0.98
P18	1.18	1.46	0.34	0.10	0.09
P19	0.36	0.87	1.70	0.00	0.48
P20	0.04	*0.11	1.76	0.18	1.10
P21	0.83	0.80	3.04	0.27	1.94
P22	1.56	1.66	0.17	0.00	0.04
P23	0.81	0.32	3.42	1.94	2.38
P24	0.79	1.57	2.67	0.31	1.22
P25	0.22	0.81	1.22	0.06	0.75
P26	1.85	2.38	2.07	0.18	0.66
P27	0.18	0.50	3.48	0.75	1.95
C01	0.05	0.01	0.11	0.05	0.01
C02	0.08	0.04	0.08	0.04	0.08
C03	0.04	0.04	0.08	0.80	0.04
C04	0.12	0.12	0.08	0.02	0.12
C05	0.10	0.10	0.08	0.02	0.14

Shown are values of OD 450 nm for each IgG autoantibody type.

aCL – anticardiolipin antibodies – Type: commercial (QUANTA Lite^®^ ACA IgG III, Inova Diagnostics). Assay Description: Purified cardiolipin bound to the wells of a polystyrene microwell plate.

aβ_2_GPI – anti-β_2_GPI antibodies – Type: commercial (QUANTA Lite^®^ ß2GP1 IgG, Inova Diagnostics). Assay Description: Purified β_2_GPI bound to the wells of a polystyrene microwell plate.

aPS/PT – anti-phosphatidylserine/prothrombin antibodies – Type: commercial (QUANTA Lite^®^ aPS/PT IgG, Inova Diagnostics). Assay Description: Plastic microwell plate wells are coated with purified PS/PT complex and then stabilized.

aPT-A – anti-prothrombin antibodies – Type: home-made (Maxisorp). Assay Description: Purified prothrombin bound to the wells of a polystyrene microwell plate.

aPT-Biot – anti-prothrombin antibodies – Type: home-made (Maxisorp). Assay Description: C-terminal biotinylated prothrombin bound to the neutravidin coated wells of a polystyrene microwell plate under conditions that preserve the antigen in its native state.

*P01 and P20 are positive for IgM aβ_2_GPI.

**Table 2:** Inter-assay variability.

	E1	E2	E3	Ave	STD	%STD
P01	0.56	0.45	0.47	0.49	0.06	12%
P03	1.42	1.40	1.63	1.48	0.12	8%
P04	1.06	0.85	1.09	1.00	0.13	13%
P06	0.62	0.45	0.57	0.55	0.09	17%
P07	1.46	1.31	1.60	1.46	0.14	10%
P09	1.34	1.29	1.52	1.38	0.12	9%
P13	0.91	0.75	0.70	0.78	0.11	14%
P14	0.84	0.78	0.90	0.84	0.06	7%
P17	0.62	0.52	0.58	0.57	0.05	9%
P19	0.49	0.45	0.45	0.46	0.02	5%
P20	0.70	0.61	0.63	0.64	0.05	7%
P21	1.24	1.13	1.41	1.26	0.14	11%
P23	1.27	1.21	1.34	1.27	0.06	5%
P24	0.57	0.45	0.52	0.51	0.06	11%
P25	0.77	0.64	0.69	0.70	0.07	9%
P26	0.70	0.64	0.63	0.66	0.03	5%
P27	1.05	1.08	1.12	1.08	0.03	3%

Shown are values of OD 450 nm measured for each patient in three different experiments (E1, E2 and E3).

IgG were used at a concentration of 50 µg/ml. ProTS525A-Biot was at 1 µg/well. ELISA experiments were repeated three times, on three different days. Each datapoint was run in triplicate. Ave, average; STD, standard deviation; % STD, Per Cent Standard deviation calculated as (STD/Ave*100).

Given that a single molecule of neutravidin can theoretically bind up to 4 molecules of biotin, we next varied the density of ProTS525A-Biot (10, 1, 0.1, 0.01 µg/well) while keeping neutravidin constant (1 µg/well). Using P27 as a source of autoantibodies, we found that the signal increased hyperbolically reaching saturation at 1 µg/well of ProTS525A-Biot (**Figure 2C**). This datapoint defines the lowest concentration of ProTS525A-Biot to achieve maximum signal. Thus, the combination 1 μg/well of neutravidin and 1 μg/well of ProTS525A-Biot, unless otherwise specified, will be used from now on in our assays.

Given that the molecular weight of prothrombin and neutravidin are comparable (72 kDa *vs* 60 kDa), the results in **Figure 2C** suggest that clustering of prothrombin driven by multivalent neutravidin, not solely antigen density, stimulates antigen-antibody complex formation. To test this hypothesis, we systematically lowered the concentration of neutravidin, from 5 µg/well to 0.25 µg/well, while keeping ProTS525A-Biot constant (1 µg/well). We observed significantly lower signal at higher neutravidin concentration (**Figure 2D**). This effect is in line with our stated hypothesis, which predicts loss of binding at high concentrations of neutravidin due to antigen spreading. Finally, to test for stability, we stored plates with bound ProTS525A-Biot for up to 2 months at 4°C. No significant differences compared to freshly prepared plates was observed.

**Figure 3:**
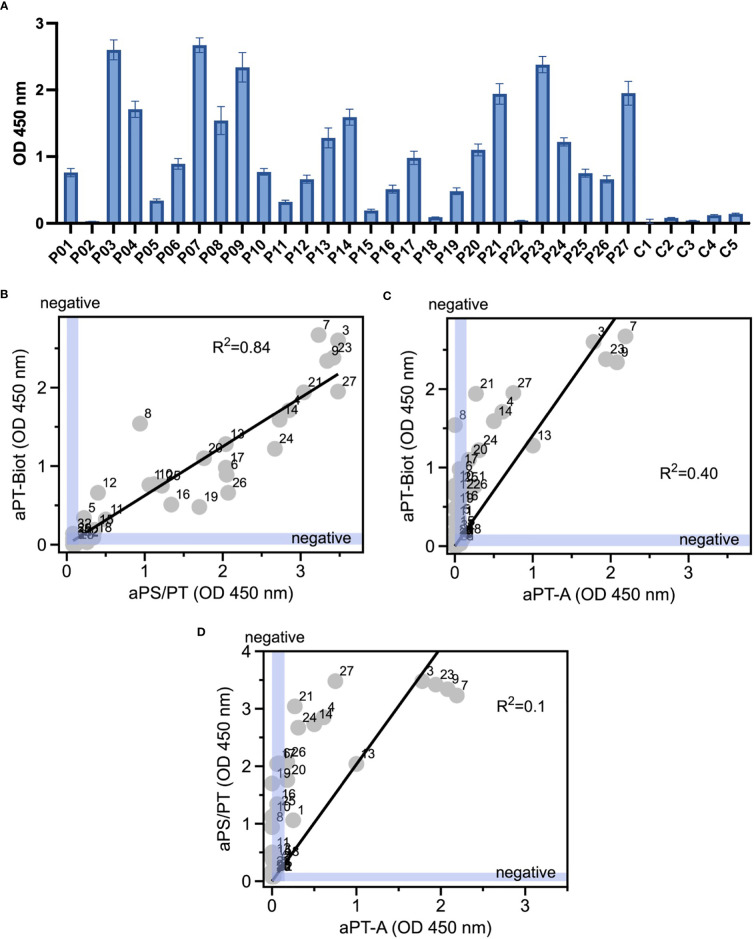
Detection of aPT-Biot in patients’ plasma and comparison with aPT-A and aPS/PT. **(A)** Detection of aPT-Biot in patients’ plasma which was diluted 1:100 v/v in TBS-B. Correlations between **(B)** aPS/PT and aPT-Biot and **(C)** aPT-A and aPT-Biot. Negative intervals for x and y axes (0<OD<0.16) are highlighted in blue. Datasets were fit with a linear regression and the coefficient of determination (R^2^) is shown in each plot. Each datapoint represents the average of three independent experiments run in duplicate. **(D)** Correlation between aPS/PT and aPT-A. Negative intervals for x and y axes are highlighted in blue. Datasets were fit with a linear regression and the coefficient of determination (R^2^) is shown. Each datapoint represents the average of three independent experiments run in duplicate.

Given the promising results with total IgG extracts, aPT-Biot were researched in a cohort of 27 triple-positive APS patients available from our previous studies (8) (P01-P27) and 5 healthy controls (C01-C05) (**Table 1**), using 1:100 v/v diluted plasma and not total IgG extracts. This design was chosen to match more closely what happens in clinical laboratories. Importantly, since all the patients fulfill the definition of triple positive APS, they are considered at high risk of developing thrombosis (17, 18). Given a cutoff of 0.16 (OD 450 nm), defined as mean OD value for healthy controls +/- 3 STD, we found 24 out of 27 (88%) APS patients positive for aPT-Biot (**Figure 3A**). When compared to aPS/PT detected using the kit manufactured by INOVA (**Figure 3B**) and aPT-A (**Figure 3C**), aPT-Bio showed an excellent linear correlation with aPS/PT (R^2^ = 0.85) but not with aPT-A (R^2^ = 0.40). Interestingly similar considerations applied when we compared aPS/PT with aPT-A (**Figure 3D**), underscoring the similarity aPT-Biot and aPS/PT and confirming the difference between aPS/PT and aPT-A discovered in earlier studies (3).

## Discussion

Developing novel, robust, and easy to interpret immunogenic assays to detect autoantibodies in patients’ plasma is a challenging yet essential task to achieve. In this study, we report the design and technical validation of a new ELISA assay for the detection of potentially pathogenic aPT in human plasma and provide initial evidence regarding its potential utility in APS patients.

One of the main problems faced by investigators when developing an ELISA assay is how to immobilize the antigen without perturbing its structural and functional properties, which are relevant for physiology and pathology. This problem needs to be considered even more carefully when antigens are inherently flexible and autoantibodies target conformational, and not linear epitopes, as is often the case for aPL (8, 19, 20). Here, we solved this issue by generating a recombinant version of prothrombin, which is site-specifically biotinylated and can be immobilized with proper orientation and desired density. Importantly, this construct retains its native structural, functional, and dynamic features in solution and when immobilized on a surface coated with neutravidin (**Figure 1**). To our knowledge, this approach is novel and has never been applied before for the detection of aPT.

In addition to successfully detecting aPT in patients’ plasma, a fascinating observation was that aPT-Biot correlated well with aPS/PT but not aPT-A. Since aPS/PT are an emerging biomarker of thrombosis in APS (4, 21) and are also found in other prothrombotic diseases such as COVID-19 (22, 23), this result is quite remarkable; it indicates that aPT-Biot may, too, find utility in the clinical practice. Rigorously designed prospective studies on a larger cohort of patients and matching controls will prove whether this idea has any merit.

Despite significant efforts and some progress, detection of aPL, and especially aPS/PT, still suffers from platform-dependent variability due to different protocols for immobilizing the antigens -which are often undisclosed due to the use of proprietary technologies- as well as the intrinsic time-dependent instability of the reagents that are immobilized onto the plastic plate (24, 25). In this context, detection of aPT-Biot provides several technical advantages compared to aPS/PT. First, provided availability of ProTS525A-Biot, this new ELISA format is very straightforward to prepare and perform, favoring transparency and reproducibility. Second, since the interaction between biotin and neutravidin is practically irreversible and calcium-independent, ELISA plates can be stored for months without loss of activity and, in principle, ELISA assays could be performed with plasma collected with a variety of anticoagulants, such as sodium citrate but also ethylenediaminetetraacetic acid (EDTA) – an interesting possibility whose validation, however, requires more experimental work. Third, since a subgroup of pathogenic aPL are known to recognize lipids and not proteins interacting with lipids (5), elimination of PS should eliminate confusions regarding which aPL are being detected.

From a biochemical standpoint, the fact that aPT-Biot correlates with aPS/PT is an intriguing finding since aPS/PT are believed to recognize cryptic epitopes resulting from the interaction of prothrombin with PS (3). While epitope mapping studies are necessary to compare similarities between these two groups of autoantibodies, because of our design and results in **Figure 3B**, aPT-Biot, like aPS/PT (8), may engage conformational epitopes that are, at least in part, contained in fragment-1. If this hypothesis is confirmed, the main role of PS could be to properly orient and concentrate the antigen on the lipid surface, thus facilitating autoantibody binding. This antigen orientation hypothesis could also help rationalize the remarkable difference between aPT-Biot and aPT-A, indicating that part of the prothrombin molecule recognized by aPT-Biot and aPS/PT is hidden or damaged when bound to hydrophilic plastic plates. However, it is also possible that aPT-Biot are different from aPS/PT and that, for example, aPS/PT might prefer epitopes that are unique in that they form when prothrombin is bound to PS, a situation that is reminiscent of recently discovered antiphospholipid antibodies targeting the endothelial protein C receptor in complex with lysobisphosphatidic acid (26).

In conclusion, we believe the new ELISA assay reported here represents an encouraging step towards a more specific detection method for aPT. Given the universality of the neutravidin-biotin system, it is also easy to envision how ProTS525A-Biot can be immobilized to other types of surfaces to enable highly reproducible and cost-effective detection of aPT in single and multiplex automated systems and to facilitate purification and characterization of aPT. Finally, a similar strategy could be applied to other antigens of aPL, such as β_2_GPI, that, despite being primed for autoantibody binding in solution (20), is currently being absorbed onto plastic plates, a harsh method resulting in unpredictable effects on its native structure. In this context, it is worth noting that encouraging results towards a more personalized diagnosis of APS have been obtained using a fragment of β_2_GPI, domain I (DI), which was either tagged at the N-terminus (27) or the C-terminus (28).

## Data Availability Statement

The original contributions presented in the study are included in the article/supplementary material. Further inquiries can be directed to the corresponding author.

## Ethics Statement

The studies involving human participants were reviewed and approved by University of Padua (VP). The patients/participants provided their written informed consent to participate in this study.

## Author Contributions

MC, VP, and NP designed, performed the research, and analyzed the data. NP wrote the manuscript. All authors contributed to the article and approved the submitted version.

## Funding

This work was supported in part by a grant R01 HL150146 (NP) from the National Heart, Lung and Blood Institute.

## Conflict of Interest

The authors declare that the research was conducted in the absence of any commercial or financial relationships that could be construed as a potential conflict of interest.

The handling editor declared a past co-authorship with one of the authors NP and declared a shared committee with the author VP.

## Publisher’s Note

All claims expressed in this article are solely those of the authors and do not necessarily represent those of their affiliated organizations, or those of the publisher, the editors and the reviewers. Any product that may be evaluated in this article, or claim that may be made by its manufacturer, is not guaranteed or endorsed by the publisher.

## References

[1] Schreiber K Sciascia S de Groot PG Devreese K Jacobsen S Ruiz-Irastorza G. Antiphospholipid Syndrome. Nat Rev Dis Primers (2018) 4:17103. 10.1038/nrdp.2017.103 29321641

[2] Meroni PL Borghi MO Raschi E Tedesco F. Pathogenesis of Antiphospholipid Syndrome: Understanding the Antibodies. Nat Rev Rheumatol (2011) 7:330–9. 10.1038/nrrheum.2011.52 21556027

[3] Sciascia S Sanna G Murru V Roccatello D Khamashta MA Bertolaccini ML. Anti-Prothrombin (aPT) and Anti-Phosphatidylserine/Prothrombin (aPS/PT) Antibodies and the Risk of Thrombosis in the Antiphospholipid Syndrome. A Systematic Rev Thromb Haemost (2014) 111:354–64. 10.1160/TH13-06-0509 24172938

[4] Cattini MG Bison E Pontara E Cheng C Denas G Pengo V. Tetra Positive Thrombotic Antiphospholipid Syndrome: Major Contribution of Anti-Phosphatidyl-Serine/Prothrombin Antibodies to Lupus Anticoagulant Activity. J Thromb Haemost (2020) 18:1124–32. 10.1111/jth.14765 32052568

[5] Lackner KJ Muller-Calleja N. Cofactor-Independent Antiphospholipid Antibodies: Implications for Pathogenesis, Diagnosis, and Treatment of Antiphospholipid Syndrome. Hamostaseologie (2019) 39:188–94. 10.1055/s-0038-1675355 30419590

[6] Chinnaraj M Chen Z Pelc LA Grese Z Bystranowska D Di Cera E. Structure of Prothrombin in the Closed Form Reveals New Details on the Mechanism of Activation. Sci Rep (2018) 8:2945. 10.1038/s41598-018-21304-1 29440720 PMC5811608

[7] Kumar S Chinnaraj M Planer W Zuo X Macor P Tedesco F. An Allosteric Redox Switch in Domain V of Beta2-Glycoprotein I Controls Membrane Binding and Anti-Domain I Autoantibody Recognition. J Biol Chem (2021) 297:100890. 10.1016/j.jbc.2021.100890 34197876 PMC8326733

[8] Chinnaraj M Planer W Pengo V Pozzi N. Discovery and Characterization of 2 Novel Subpopulations of aPS/PT Antibodies in Patients at High Risk of Thrombosis. Blood Adv (2019) 3:1738–49. 10.1182/bloodadvances.2019030932 PMC6560356 31175129

[9] Chinnaraj M Barrios DA Frieden C Heyduk T Flaumenhaft R Pozzi N. Bioorthogonal Chemistry Enables Single-Molecule FRET Measurements of Catalytically Active Protein Disulfide Isomerase. Chembiochem (2021) 22:134–8. 10.1002/cbic.202000537 PMC7790914 32857455

[10] Pozzi N Bystranowska D Zuo X Di Cera E. Structural Architecture of Prothrombin in Solution Revealed by Single Molecule Spectroscopy. J Biol Chem (2016) 291:18107–16. 10.1074/jbc.M116.738310 PMC5000060 27435675

[11] Schrimpf W Barth A Hendrix J Lamb DC. PAM: A Framework for Integrated Analysis of Imaging, Single-Molecule, and Ensemble Fluorescence Data. Biophys J (2018) 114:1518–28. 10.1016/j.bpj.2018.02.035 PMC5954487 29642023

[12] Singh SP Soranno A Sparks MA Galletto R. Branched Unwinding Mechanism of the Pif1 Family of DNA Helicases. Proc Natl Acad Sci USA (2019) 116:24533–41. 10.1073/pnas.1915654116 PMC6900637 31744872

[13] Chandradoss SD Haagsma AC Lee YK Hwang JH Nam JM Joo C. Surface Passivation for Single-Molecule Protein Studies. J Vis Exp (2014). 10.3791/50549 PMC4179479 24797261

[14] Bronson JE Fei J Hofman JM Gonzalez RL Jr. Wiggins CH. Learning Rates and States From Biophysical Time Series: A Bayesian Approach to Model Selection and Single-Molecule FRET Data. Biophys J (2009) 97:3196–205. 10.1016/j.bpj.2009.09.031 PMC2793368 20006957

[15] Miyakis S Lockshin MD Atsumi T Branch DW Brey RL Cervera R. International Consensus Statement on an Update of the Classification Criteria for Definite Antiphospholipid Syndrome (APS). J Thromb Haemost (2006) 4:295–306. 10.1111/j.1538-7836.2006.01753.x 16420554

[16] Pozzi N Chen Z Zapata F Niu W Barranco-Medina S Pelc LA. Autoactivation of Thrombin Precursors. J Biol Chem (2013) 288:11601–10. 10.1074/jbc.M113.451542 PMC3630838 23467412

[17] Banzato A Pozzi N Frasson R De Filippis V Ruffatti A Bison E. Antibodies to Domain I of Beta(2)Glycoprotein I are in Close Relation to Patients Risk Categories in Antiphospholipid Syndrome (APS). Thromb Res (2011) 128:583–6. 10.1016/j.thromres.2011.04.021 21620443

[18] Chayoua W Kelchtermans H Moore GW Musial J Wahl D de Laat B. Identification of High Thrombotic Risk Triple-Positive Antiphospholipid Syndrome Patients is Dependent on Anti-Cardiolipin and Anti-Beta2glycoprotein I Antibody Detection Assays. J Thromb Haemost (2018) 16:2016–23. 10.1111/jth.14261 30079628

[19] de Laat B Derksen RH van Lummel M Pennings MT de Groot PG. Pathogenic Anti-Beta2-Glycoprotein I Antibodies Recognize Domain I of Beta2-Glycoprotein I Only After a Conformational Change. Blood (2006) 107:1916–24. 10.1182/blood-2005-05-1943 16269621

[20] Ruben E Planer W Chinnaraj M Chen Z Zuo X Pengo V. The J-Elongated Conformation of Beta2-Glycoprotein I Predominates in Solution: Implications for Our Understanding of Antiphospholipid Syndrome. J Biol Chem (2020) 295:10794–806. 10.1074/jbc.RA120.013939 PMC7397106 32518155

[21] Pengo V Del Ross T Ruffatti A Bison E Cattini MG Pontara E. Lupus Anticoagulant Identifies Two Distinct Groups of Patients With Different Antibody Patterns. Thromb Res (2018) 172:172–8. 10.1016/j.thromres.2018.11.003 30466070

[22] Borghi MO Beltagy A Garrafa E Curreli D Cecchini G Bodio C. Anti-Phospholipid Antibodies in COVID-19 Are Different From Those Detectable in the Anti-Phospholipid Syndrome. Front Immunol (2020) 11:584241. 10.3389/fimmu.2020.584241 33178218 PMC7593765

[23] Zuo Y Estes SK Ali RA Gandhi AA Yalavarthi S Shi H. Prothrombotic Autoantibodies in Serum From Patients Hospitalized With COVID-19. Sci Transl Med (2020) 12(570):eabd3876. 10.1126/scitranslmed.abd3876 33139519 PMC7724273

[24] Willis R Pierangeli SS Jaskowski TD Malmberg E Guerra M Salmon JE. Performance Characteristics of Commercial Immunoassays for the Detection of IgG and IgM Antibodies to Beta2 Glycoprotein I and an Initial Assessment of Newly Developed Reference Materials for Assay Calibration. Am J Clin Pathol (2016) 145:796–805. 10.1093/ajcp/aqw065 27267373

[25] Pengo V Bison E Denas G Jose SP Zoppellaro G Banzato A. Laboratory Diagnostics of Antiphospholipid Syndrome. Semin Thromb Hemost (2018) 44:439–44. 10.1055/s-0037-1601331 28470652

[26] Muller-Calleja N Hollerbach A Royce J Ritter S Pedrosa D Madhusudhan T. Lipid Presentation by the Protein C Receptor Links Coagulation With Autoimmunity. Science (2021) 371. 10.1126/science.abc0956 PMC9014225 33707237

[27] Pozzi N Banzato A Bettin S Bison E Pengo V De Filippis V. Chemical Synthesis and Characterization of Wild-Type and Biotinylated N-Terminal Domain 1-64 of Beta2-Glycoprotein I. Protein Sci (2010) 19:1065–78. 10.1002/pro.387 PMC2868248 20440842

[28] Ioannou Y Giles I Lambrianides A Richardson C Pearl LH Latchman DS. A Novel Expression System of Domain I of Human Beta2 Glycoprotein I in Escherichia Coli. BMC Biotechnol (2006) 6:8. 10.1186/1472-6750-6-8 16472380 PMC1402286

